# *Mycobacterium avium* Subsp.* paratuberculosis* Induces Specific IgE Production in Japanese People with Allergies

**DOI:** 10.1155/2017/7959154

**Published:** 2017-04-24

**Authors:** D. Cossu, S. Otsubo, Y. Otsubo, S. Eda, T. Suzuki, Y. Iwao, T. Kuribayashi, S. Yamamoto, L. A. Sechi, E. Momotani

**Affiliations:** ^1^Department of Human Care, Tohto College of Health Sciences, Fukaya, Saitama, Japan; ^2^Sangenjaya Hospital, Setagaya, Tokyo, Japan; ^3^Center for Wildlife Health, Department of Forestry, Wildlife and Fisheries, The University of Tennessee, Knoxville, TN, USA; ^4^Laboratory of Immunology, School of Life and Environmental Science, Azabu University, Sagamihara, Kanagawa, Japan; ^5^Department of Biomedical Sciences, Division of Microbiology and Virology, University of Sassari, Sardinia, Italy

## Abstract

*Background*. The prevalence of allergies is steadily increasing worldwide; however, the pathogenesis is still unclear. We hypothesized that* Mycobacterium avium* subsp.* paratuberculosis* (MAP) may contribute to allergy development. This organism can be present in dairy foods, it can elicit an immunomodulatory switch from a Th1 to a Th2 response, and it has been speculated that it is linked to several human autoimmune diseases. To determine the contribution, sera from 99 individuals with various atopic disorders and 45 healthy nonallergic controls were assessed for total IgE levels and successively for MAP-specific IgE by ELISA.* Results*. The mean total serum IgE level in allergic patients was 256 ± 235 IU/mL, and in the healthy controls it was 62 ± 44 IU/mL (AUC = 0.88; *p* < 0.0001). Among the patient groups, 50 of the 99 subjects had increased IgE total level ≥ 150 IU/mL, while 49 subjects had IgE ≤ 150 IU/mL (mean level: 407 ± 256 IU/mL versus 106 ± 16 IU/mL; *p* < 0.0001). Additionally, 6 out of 50 subjects (12%) with IgE ≥ 150 IU/mL and none (0%) with IgE ≤ 150 IU/mL were positive for specific MAP IgE (AUC = 0.63; *p* = 0.03).* Conclusion.* The present study revealed that MAP has the ability to induce specific IgE and might contribute to the induction of allergic inflammation in genetically predisposed individuals.

## 1. Background

There is increased prevalence of diseases due to immunological disorders including autoimmune diseases and allergies in developed countries [1, 2]. It has been believed that only the changing of environmental exposures can modulate the human immune system, but the trigger factor has not yet been clarified. Allergic diseases have common causative and inducible factors, including the environment, genetic predisposition [3, 4], mucosal barrier dysfunction [5], and alteration of commensal microbiota [6, 7]. Allergy is considered to be a dysfunction of immunological tolerance. In experiments with animal models of allergy, several adjuvants such as complete Freund's adjuvant, aluminum hydroxide [8], cholera toxin [9], and zymosan [10], as well as the allergy adjuvant effect of particles [11], have been considered as essential factors for the induction of allergy. It is well known that mycobacteria and their components have been the basis of numerous adjuvants and immunotherapies; in particular complete Freund's adjuvant is used to elicit strong humoral and cellular immune responses in experiments with animals [12]. Since there have been no studies to date on the role of mycobacteria in the pathogenesis of human allergies, we evaluated the allergenicity of* Mycobacterium avium* subspecies* paratuberculosis* (MAP) and its possible contribution to the pathogenesis of these diseases.

MAP is an intracellular pathogen that causes paratuberculosis (Johne's disease) in ruminants, a chronic granulomatous enteritis causing major economic losses in the dairy industry worldwide [13].

Due to the pathological similarities between paratuberculosis and human Crohn's disease (CD), a chronic inflammatory bowel disease of the gastrointestinal tract, MAP has long been suspected to be the etiological agent of CD due to frequent detection of MAP-specific DNA and its infrequent isolation from the lesion [14]. Experimental CD-type colitis induced with the MAP antigen also suggests the potency of MAP to induce CD [4]. MAP was also linked with others autoimmune diseases such as type 1 diabetes, multiple sclerosis, and Hashimoto's thyroiditis [15–18].

The shedding of MAP into milk is well known in the infected cattle, and there is much evidence of MAP contamination in milk, cheese, powdered milk for infants, and beef meat [19, 20]. The bacilli can also survive the pasteurization process [21]. Thus the existence of a food-born route of MAP exposure from animals to human is likely [20]. A recent seroprevalence study demonstrated the presence of an immunoglobulin G (IgG) subclass pattern against MAP lipophilic antigens [20] in healthy Japanese people. Of particular interest, the detection of IgG4 provides evidence of prolonged antigenic stimulation [22].

Moreover, oral exposure of mycobacteria is important in the induction of intestinal tuberculosis in human and paratuberculosis in cattle. Recent vaccination studies for the control of tuberculosis in wild animals showed that oral administration of heat-killed mycobacteria stimulating host immunity [23, 24].

Based on this evidence, we hypothesized that oral exposure to MAP antigens might be able in turn to modulate host immunity enabling the development of allergies. In the present study, we examined the sera of allergic individuals and healthy controls (HCs) for the presence of MAP-specific IgE in the context of total IgE levels. To our knowledge, this is the first report showing greater presence of MAP-specific IgE in the serum of allergic individualswith higher levels of total IgE compared to nonallergic people.

## 2. Materials and Methods

### 2.1. Participants and Samples

A total of 99 subjects with reported allergy (F/M = 74/25; mean age: 41.65 ± 11.5) and 45 age- and sex-matched HCs without allergy (F/M = 31/14; mean age: 34.23 ± 3.4) were enrolled from healthcare personnel of Sangenjaya hospital, Tokyo, from March to April 2014. All subjects were interviewed using questionnaires and gave their written informed consent. Many of them had a history of sensitivity to allergenic food and/or common aeroallergens. The demographic and clinical characteristics of the patients are reported in Table 1. All participants recruited in the present study were ethnically Japanese for at least three generations and did not use any kind of immunosuppressive drugs prior to sampling. Five milliliters of heparinized blood sample was taken from each participant. After separation by centrifugation, the sera were stored at −20°C until testing by ELISA.

The study protocols, the documents for informed consent, and the questionnaire sheets were approved by the Human Ethics Committee of Sangenjaya Hospital (approval number: 2014.3.7) and Tohto College of Health Sciences (approval number: H2511).

### 2.2. Antigens

Preparation of MAP (strain ATCC 19698) and* Bacillus Calmette-Guérin* (BCG) (strain Tokyo 172) lipophilic antigen was performed as previously published [20]. MAP lipophilic antigen is an ethanol (crude) extract of MAP and its antigenicity is due to the lipid (chloroform extractable) and not to the protein (aqueous) fraction [25]. An analysis of the lipid fraction carried out by thin-layer chromatography showed that it contained MAP-specific lipids, and one of these is the major cell wall-associated MAP-specific lipopeptide Para-LP-01 [26]. The latter, also called lipopentapeptide (L5P), is capable of inducing a strong host humoral response and distinguishes MAP from* Mycobacterium avium* subsp.* hominissuis* and* Mycobacterium avium* subsp.* avium* [27].

### 2.3. Measurement of Total and MAP-Specific Serum IgE by ELISA

The commercially available Human IgE ELISA Quantitation Set Kit (Bethyl Laboratories Inc., Montgomery, TX, USA) was used to detect human IgE in the sera according to the manufacturer's protocol. The test kit provides an affinity purified goat anti-human IgE antibody (Ab), a standard, and a horseradish peroxidase (HRP) conjugated goat anti-human IgE detection Ab (used at a dilution of 1 : 40,000). Samples and standards were run in duplicate.

Serum specific IgE to MAP lipophilic antigen were measured through indirect ELISA [25]. The absorption of human sera with* Mycobacterium phlei* prior to testing was of value in eliminating falsely positive reactions in ELISA for the detection of Abs against MAP [28]. Indeed this step partially removes cross-reacting Abs and it provides high specificity [28].

Sumilon ELISA 96-well plates (MS-8496F, Sumitomo Bakelite Co., Ltd., Tokyo, Japan) were coated with 50 *μ*L/well of diluted lipophilic antigens at room temperature overnight. The next day, blocking was performed with 300 *μ*L/well of Blocking One solution (Nacalai Tesque, Kyoto, Japan) with incubation at room temperature for one hour. The plates were washed three times with 300 *μ*L/well PBST (10 mM phosphate-buffered saline, pH 7.0, and 0.5% Tween 20). Duplicates were made on the 96-well plate for each sample. Then 100 *μ*L/well of preabsorbed serum sample at a 1 : 100 dilution in diluting buffer (PBST) was then added and incubated at room temperature for two hours, followed by an additional washing step. HRP-labeled goat anti-human IgE polyclonal Ab (SeraCare Life Sciences, Gaithersburg, MD, USA) (1 : 1000 dilution) was added and incubated at room temperature for two hours, followed by a washing step. The bound HRP conjugate was detected by adding 100 *μ*L/well of Sure Blue Reserve TMB Microwell Peroxidase Substrate (SeraCare Life Sciences, Gaithersburg, MD, USA) and the absorbance was measured at a wavelength of 450 nm by using a Bio-Rad iMark Microplate Reader (Bio-Rad, Tokyo, Japan). Data were normalized to a positive control serum included in all assays run, whose Ab reactivity was set at 10,000 arbitrary units (AU)/mL. A negative control (antigen and secondary Ab alone) was also included and the mean values were subtracted from all the samples.

### 2.4. Inhibition Assay

To evaluate the specificity of MAP antigens in stimulating an Abs response and to verify whether cross-recognition between MAP and other mycobacteria occurs, an inhibition ELISA using antigen of BCG was developed. BCG belongs to the* Mycobacterium tuberculosis* complex, far from the* Mycobacterium avium* complex, and concerns other routes of exposition. In any case, all the subjects enrolled in the study were BCG-vaccinated and MAP shares different antigens with BCG [29]; this may lead to false positive reactions.

An inhibition assay was performed using two serum pools (each pool, tested in two separate experiments, was composed of three sera of anti-MAP IgE+/IgG− subjects) as previously reported [8]. Briefly, plates were coated with MAP lipophilic antigen and incubated at room temperature overnight. Inhibition assays were performed by preincubating the serum pools overnight at 4°C with saturating concentrations of BCG antigens and MAP antigens (added as positive control). The following day, the serum pools were subjected to ELISA on plates coated with MAP lipophilic antigen. Subsequent steps were identical to the indirect ELISA for detection of Abs as described above.

### 2.5. Statistical Analysis

GraphPad Prism 6.0 statistical software (La Jolla, CA, USA) was used for analysis of the data. Fisher's exact test with Yates' corrections was used to compare the mean total serum IgE levels between subjects with total IgE of ≥ 150 IU/mL (considered pathological) and ≤ 150 IU/mL (considered normal or borderline range) and to compare IgE levels between allergic patients and HCs.

Concerning the specific MAP IgE titer, for comparison between the groups we used Mann–Whitney *U* test. All statistical tests were two-sided with a significance level of 0.05 and all results are reported as the median ± interquartile range. The optimum cutoff values were calculated by a Receiver Operating Characteristic (ROC) curves analysis, setting the specificity at 95%, with the corresponding sensitivity chosen accordingly. The odds ratios (OR) with a 95% confidence interval (CI) were also calculated.

## 3. Results

### 3.1. Total IgE Ab Serum Levels

**Figure 1:**
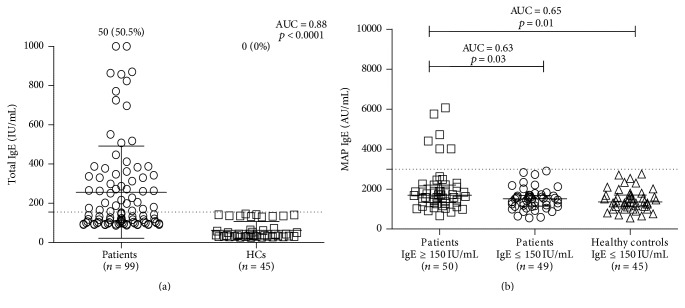
Levels of total and MAP-specific serum IgE in Japanese subjects. (a) Total IgE: data are expressed in IU/mL, including the mean and the standard deviation. Percentages of samples with IgE > 150 IU/mL are indicated at the top of distribution. (b) MAP-specific IgE: data are expressed in AU/mL, and the median ± interquartile range is indicated by horizontal bars. The dotted lines indicate the cutoff of the reactions. AUC, area under ROC curve.

The mean total serum IgE level in the allergic patients was 256 ± 235 IU/mL, whereas in the HCs it was 62 ± 44 IU/mL (AUC = 0.88; *p* < 0.0001) (Figure 1(a)). Fifty of the 99 patients (F/M = 40/10; mean age: 42.8 ± 12.3) studied had an increased IgE total level ≥ 150 IU/mL, while 49 subjects (F/M = 34/15; mean age: 40.5 ± 10.8) had an IgE of ≤150 IU/mL. The mean total serum IgE level in patients with high IgE levels was 407 ± 256 IU/mL, while in subjects with low IgE levels it was 106 ± 16 IU/mL (*p* < 0.0001).

Total IgE serum levels were negatively associated with age/gender. In contrast, total IgE serum levels were associated with sensitivity to different allergens. Of the 50 subjects with high total IgE level of ≥150 IU/mL, 21 (42%) were sensitive at least to one allergenic food (mackerel, corned food, egg white, milk, mango, shrimp, crab, and wheat) (OR = 1.2; 95% CI: 0.5–2.8). Twenty-five subjects (50%) were sensitive to aeroallergen, 15 to pollen of Japanese cedar (OR = 1.2; 95% CI: 0.5–2.8), and 10 to Japanese cypress (OR = 0.97; 95% CI: 0.4–2.6), while four (8%) had allergy for both food and inhalant allergens (OR = 1.3; 95% CI: 0.3–6.3). In addition, 40 subjects (80%) habitually consumed imported products (OR = 3; 95% CI: 1.2–7.3).

### 3.2. Detection of MAP-Specific IgE in Subjects with High Total IgE Levels

Regarding the humoral response against MAP-specific antigen, we tested all allergic subjects taking into account their total serum IgE level. As shown in Figure 1(b), 6 out of 50 (12%) subjects with an IgE of ≥ 150 IU/mL and 0 out of 49 (0%) with an IgE of ≤ 150 IU/mL were positive for specific anti-MAP IgE. The association between the two groups is considered statistically significant (AUC = 0.63; *p* = 0.03). The mean total serum IgE level in these six patients was 472 ± 237 IU/mL. MAP-specific IgE Abs were not detected (Figure 1(b)) in HCs group; the association between the allergic subjects with IgE ≥ 150 IU/mL and HCs is statistically significant (AUC = 0.65; *p* = 0.01).

Among the six positive subjects, five were female and one was male (OR = 1.3; 95% CI: 0.1–12). Four were allergic to Japanese cedar* (Cryptomeria japonica)* and two to Japanese cypress* (Chamaecyparis obtusa)* (OR = 1.3; 95% CI: 0.2–10). Note that all of the positive subjects preferred to consume imported cheese and milk, and two of them were also allergic to milk.

Concerning anti-MAP IgG titer (previously reported) [8], double positivity (anti-MAP IgE and anti-MAP IgG) was found in one out of six anti-MAP IgE positive patients; thus we cannot deny the possibility of IgG competition or the presence of IgG autoantibodies to IgE in this subject [30].

The interassay coefficient of variation (CV) (with the same serum tested in three separate experiments) for the indirect ELISAs ranged from 5.4% to 8.5%, while the intra-assay CV (with the same serum tested in 20 replicate wells) ranged from 1.9% to 3.3%.

### 3.3. Anti-MAP and Anti-BCG Antibodies Targeting Lipophilic Antigens Are Not Cross-Reactive

**Figure 2:**
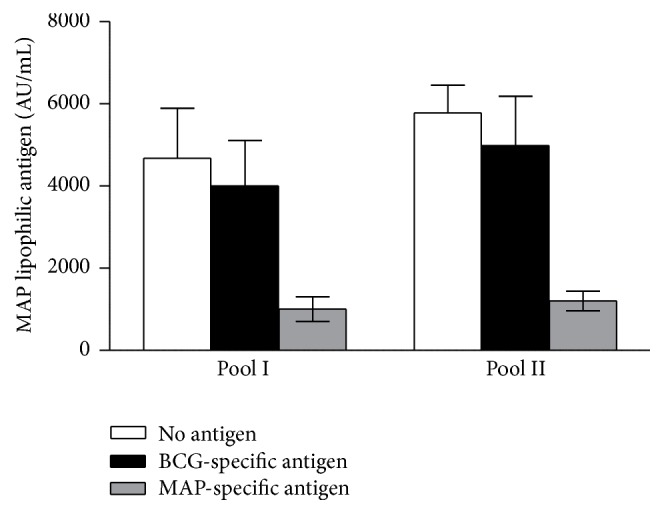
Inhibition ELISA results showing the specificity of MAP antigens in serum pools of subjects. Two serum pools of subject MAP IgE+/IgG− were preincubated overnight in plates coated with MAP lipophilic antigen with saturating concentrations of BCG antigen and MAP antigen and no antigen (a regularly performed ELISA at 1/100 serum dilution). The results are representative of two separate experiments.

Next, we determined the presence or absence of cross-recognition between MAP and BCG by inhibition ELISA using two serum pools. Regarding the specificity of MAP lipophilic antigens, we confirmed by inhibition experiments that anti-MAP and anti-BCG Abs targeting surface lipophilic antigen are not cross-reactive. Indeed, for both serum pools tested, BCG antigen was not capable of significantly blocking the Ab-binding directed to MAP antigens, causing only a small decline in the signal (11–15%), while MAP antigen (positive control) inhibited the signal of all serum pools tested (79–82%) (Figure 2).

## 4. Discussion

The present study revealed that Japanese people have anti-MAP-specific IgE and that positive individuals had respiratory allergy with higher levels of total IgE. We detected statistically significant high levels of specific anti-MAP IgE in six subjects with total serum IgE of ≥150 IU/mL and in none of the subjects with IgE of ≤150 IU/mL. This surprising result shows for the first time that MAP antigens are able to elicit a specific IgE response in human. This evidence may provide a new view of pathogenesis of allergy increasing worldwide [2]. Considering that the incidence of bovine paratuberculosis and the level of contamination with MAP antigen in dairy products are growing year by year [31], there is also the possibility that oral exposure to MAP antigens can increase. Since the incidence of bovine paratuberculosis is very low in Japan, the Japanese population could be exposed to MAP via contaminated dairy products imported from Western countries, where the prevalence of paratuberculosis is very high [20].

In our study, all of the IgE positive subjects were affected by allergies caused by aeroallergens and often consumed imported dairy products. Hence, it is possible that these individuals are regularly exposed to allergens to the same degree that they are exposed to MAP antigens orally.

The prevalence of allergic disorders is steadily increasing worldwide [2] including in Japan [32]. This is probably attributable to weakened immunologic tolerance or enhanced reaction to environmental antigens [33]. The role of the environment in influencing allergies is still an open topic, and it is unclear which kind of immunoregulatory mechanism can occur through interaction between pathogenic environmental antigenic stimuli and the immune system [33].

MAP is reported to have a significantly stronger ability to induce IL-10 in a host macrophage compared with other mycobacteria [34–37]. MAP polymorphic PPE proteins have a stronger capability of inducing IL-10 expression in the host, thus suppressing the production of Th1-related cytokines such as interferon gamma [21] and enhancing Th2 reaction (IL-4) expression. These MAP antigens are considered to be a weapon to impair the most powerful host cellular immunological defenses and to achieve chronic infection in cattle [35]. The phenomenon that stimulates host Th1 and Th2 in different stages of infection [18] is very similar to that in human leprosy [38, 39].

Another possible link between MAP and allergies is based on a molecular mimicry mechanism. In fact, Blast-p analysis revealed that the major antigenic peptide from the Japanese cedar pollen allergen Cry j 1 (KVTVAFNQF) [40] shares its sequence homology with several MAP hydroxylase proteins. These proteins present six conserved amino acids residues (TVALNQF) identical to Cry j 1, with a sequence identity of 86%. A hypothetical explanation is that MAP antigenic components might cross-react with themselves or with foreign antigens that the host would be in almost permanent contact with, and this could alter the regulatory state of the host in the absence of a MAP infection. Indeed, during the immune response against this obligate intracellular pathogen, there might be an expansion of autoreactive regulatory cells, such as FoxP3+ or FoxP3− Treg cells [41], which could be constantly activated by autoantigens, even if the bacilli are cleared by the natural immune responses [42]. We note that several examples of cross-reactivity of MAP with human autoantigens have been reported [16–18].

In conclusion, despite the fact that mycobacterial antigen is generally considered to downregulate the IgE response by normalizing the balance of Th1 and Th2 [43], our data demonstrate that MAP antigens are capable of inducing specific IgE production. MAP could play a role in IgE-mediated allergic inflammation due to (1) the adjuvant function of mycobacterial antigen [3], (2) its IL-10 inducible nature, and (3) continuous immune stimulation by antigens shared with foods. The consumption of MAP-contaminated products could be the vehicle for the exposure of MAP antigen to human.

## 5. Conclusion

The present study revealed that MAP has the ability to induce specific IgE and might contribute to the induction of allergies in genetically predisposed individuals.

## Figures and Tables

**Table 1:** Demographic and clinical characteristics of the allergic patients.

Patients (*n* = 99)	IgE ≥ 150 IU/mL (*n* = 50)	IgE ≤ 150 IU/mL (*n* = 49)	MAP specific IgE (*n* = 6)
Mean age	42.8 ± 12.3	40.5 ± 10.8	39.2 ± 10.4
Gender			
Female	40 (80%)	34 (69%)	5 (83%)
Male	10 (20%)	15 (31%)	1 (17%)
History of allergic diseases			
Food allergy^*∗*^	21 (42%)	18 (37%)	2 (33%)
Respiratory^*∗∗*^	25 (50%)	23 (47%)	6 (100%)
Daily products consumption	40 (80%)	18 (37%)	6 (100%)
Autoimmune diseases^**∗∗∗**^	2 (4%)	4 (8%)	0 (0%)
Infections			
*M. tuberculosis*	0 (0%)	0 (0%)	0 (0%)
Nontuberculous mycobacteria	0 (0%)	0 (0%)	0 (0%)
BCG vaccination	50 (100%)	49 (100%)	6 (100%)
Total IgE levels (IU/mL)	407 ± 256	106 ± 16	472 ± 237

^*∗*^Mackerel, corned food, egg white, milk, mango, shrimp, crab, and wheat.

^*∗∗*^Japanese cedar and Japanese cypress.

^*∗∗∗*^Inflammatory bowel disease and type 1 diabetes.
